# Addressing the shortage of pathologists in Africa: Creation of a MMed Programme in Pathology in Zambia

**DOI:** 10.4102/ajlm.v9i1.974

**Published:** 2020-06-03

**Authors:** Victor Mudenda, Evans Malyangu, Shahin Sayed, Kenneth Fleming

**Affiliations:** 1 Department of Pathology and Microbiology, University Teaching Hospital, Lusaka, Zambia; 2 Department of Pathology, Maina Soko Military Hospital, Lusaka, Zambia; 3 Department of Pathology, Aga Khan University Hospital, Nairobi, Kenya; 4 Green Templeton College, University of Oxford, Oxford, United Kingdom

**Keywords:** pathologist shortage, Africa, Postgraduate MMed, Zambia, College of Pathologists of East Central and Southern Africa (COPECSA)

## Abstract

**Background:**

With approximately one pathologist for one million people compared to ratios of approximately 1 to 25 000 in the United States and United Kingdom, there is a severe shortage of pathologists in much of Africa. The situation is particularly severe in Zambia, where, in 2009, the ratio was 1 to 1.4 million.

**Objective:**

To address this, a postgraduate Master of Medicine (MMed) training programme was launched in Lusaka in 2011.

**Methods:**

The process and most significant challenges and lessons learned were documented, as they may be of value to other countries facing similar challenges.

**Results:**

Since 2011, four Zambian pathologists have graduated, doubling the number of indigenous pathologists in the country. Currently 10 students are in training. The most significant problem was issues arising from the split responsibilities of the Ministries of Health and of Education and the most important lesson learned was the crucial need for broad local ownership and commitment.

**Conclusion:**

Successfully addressing the shortage of local pathologists by creating country-specific, postgraduate MMed training programmes, even in situations of restricted resources, is feasible. However, having access to and support from the shared resources, expertise and knowledge of a regional College of Pathologists would be a major advantage.

## Introduction

Although hard data are sparse, it has become increasingly recognised over the last decade that there is grossly inadequate capacity in pathology and laboratory medicine in many low- and middle-income countries (LMICs), especially in sub-Saharan Africa, and that, even where present, standards are highly variable.^1^ Addressing this issue will require multi-year and multifaceted approaches, especially given both the increasing population and the increasing burden of non-communicable diseases facing many LMICs.^2^

Zambia has a population of around 17 million. In 2000, it was one of the poorest countries in the world, but over the next decade, rapid growth raised it into the lower middle-income bracket (https://data.worldbank.org/country). It has a high infectious disease burden, but it has also seen a rise in non-communicable diseases, such as ischaemic heart disease and cancer (especially HIV-related cancers) (http://www.healthdata.org/zambia). Given the shortage of doctors and other healthcare workers needed to face this double healthcare challenge, in 2009, both the 5th National Development Plan and the Ministry of Health National Strategic Plan^3^ identified human resource development as critical to meeting the United Nations Millennium Development Goals for Health (https://www.un.org/millenniumgoals/).

A particular problem was the shortage of pathologists, with the resultant lack of medical input into laboratory diagnosis and management of disease. Pathologists are medically qualified specialists in all the clinical laboratory disciplines – haematology, microbiology, clinical chemistry, immunology, anatomic pathology, etc. While non-medical laboratory scientists can also provide laboratory services, they do not do so in anatomic pathology. Anatomic pathology is concerned with diagnosis based on morphology of the tissues, using cytology, microscopy and autopsy. As tissue diagnosis is the gold standard for many diseases, including all cancers, and many infectious and non-infectious diseases of, for example, liver, kidney, gastro-intestinal tract, muscle and nerve, it is crucial for accurate therapy and management. It also reduces errors and resultant health costs and improves accuracy of health data for planning and budgeting (health management and financing).

The shortage was partly due to the general lack of financing for medical education, but also reflected the lack of local faculty to provide the specialist training. In 2009, there were fewer than 10 anatomic pathologists working in Zambia (of whom only 4 were Zambian), a population ratio of 1 to 1.4 million. For comparison, in the United Kingdom, there are about 1800 anatomic pathologists for a population of about 66 million, a population ratio of 1 to 36 000. This deficit meant the majority of the population had very limited access to pathology expertise. To illustrate this, in 2009, the main pathology department in the country, based in the University Teaching Hospital in Lusaka, population around 1.7 million, had around 5000 anatomic pathology cases per year. In contrast, Oxford, United Kingdom, which serves an immediate population of around 750 000 and a wider population of around 2.5 million (incorporating five district general hospitals), has around 55 000 anatomic pathology cases per year (personal information, K.F.). In addition, prior to 2007, cancer therapy had been largely provided in South Africa or Zimbabwe. However, the opening of the Cancer Diseases Hospital in Lusaka in 2007 made provision of appropriate local anatomic pathology services crucial. Furthermore, since 2004, the University of Zambia had trained an increasing number of laboratory scientists, but without a parallel increase in pathologists.

Because there was no local pathologist training programme, small numbers of pathologists had been trained abroad (e.g. United Kingdom, South Africa), but the great majority had not returned to Zambia. Accordingly, in 2009, in recognition of the need to increase clinical input into laboratory diagnoses and address the shortfall in anatomic pathology, the Ministry of Health identified the local training of pathologists as one of its priorities. To do this, it was decided to set up a Master of Medicine (MMed) degree programme in Pathology at the School of Medicine at University of Zambia. The salary costs of the trainees and of the local trainers and some local infrastructure would be covered by the university and hospital. But given the shortage of specialist staff, equipment and other infrastructure, assistance to set up and run the programme was sought from the Tropical Health Education Trust, London. A grant from the Department for International Development, United Kingdom, to the Tropical Health Education Trust (THET) funded the recruitment and travel of external trainers, infrastructure, such as microscopes, books, modest refurbishment of space and the costs of the external placements of the students.

As there are very few publications describing the creation of pathologist training programmes in Africa,^4,5,6^ and as many other LMICs have a similar lack of pathologists as in Zambia, exploring the problems the Zambia MMed faced and overcame, and describing the lessons learned from this process, may act as a roadmap for such countries. Furthermore, the launch of this programme in 2011 coincided with the launch of the College of Pathologists for East, Central and Southern Africa (COPECSA) (see accompanying article^7^). As many of the issues identified in Zambia illustrate the potential role for such colleges to assist LMICs in improving the quantity and quality of training in relevant specialties, this article also illustrates how the Zambia MMed could have benefited from the prior existence of COPECSA.

## Intervention

### Creation of the Master of Medicine programme

In 2010, a needs assessment was conducted in Lusaka to analyse the current situation, determine the magnitude of the need and plan the development of a MMed Programme in Pathology. The assessment took into account the major concerns of the public and other stakeholders and health workers, especially clinicians. To do this there were internal interviews with lecturers and students at University of Zambia and key stakeholders including clinicians and Ministry of Health staff, meetings within other clinical departments across the school, and input from a small number of United Kingdom pathologists.

Throughout this process, there was considerable discussion about duration and content of the curriculum. Proposals ranged from a one-year or two-year multidisciplinary programme involving haematology, microbiology, clinical biochemistry and anatomic pathology, to a four-year mono-specialty course in cell pathology, with variants in between. The argument for the former was that as the greatest need was for service provision in all the disciplines of pathology, particularly outside the capital city, producing significant numbers of multi-specialty staff as quickly as possible should therefore be the priority. These could be either medical or non-medical staff. Conversely, it was argued that as the ‘clinical pathology’ specialties of microbiology, biochemistry and haematology already had some modest provision through scientists with Bachelor of Science (BSc), Master of Science (MSc), or Doctor of Philosophy (PhD) degrees, the absolute crucial need was for medically qualified anatomic pathologists. As the Ministry of Health had ordained that the objective of the programme was to produce pathologists who would direct provincial hospital laboratories, with overall responsibility for all aspects of pathology, but with personal responsibility for anatomic pathology, eventually the university and ministry agreed that a four-year course, predominantly focused on anatomic pathology, but with some involvement in haematology, microbiology and clinical biochemistry, would be adopted.

**FIGURE 1:**
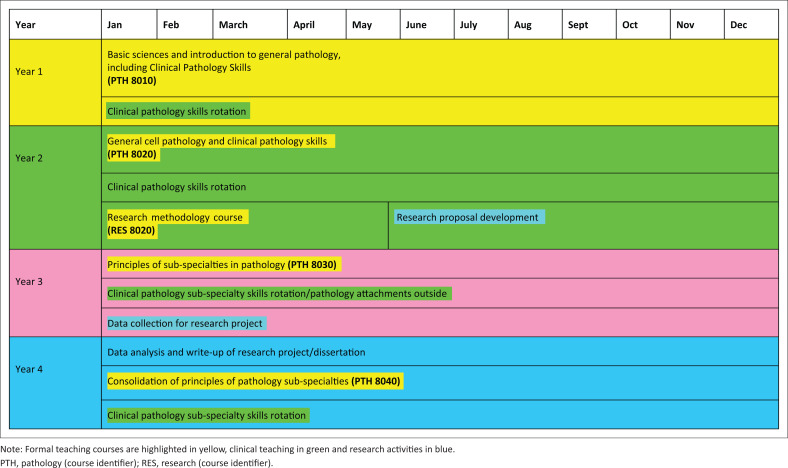
Programme map for development of MMed programme, Zambia, 2010–2016.

The process of discussion culminated in a two-day meeting in 2010 for the definition of the aims and objectives of the course, development of a curriculum, determination of the modes of teaching and agreement on the modes of evaluation of the students (see Figure 1). To encourage retention, students would be bonded for the duration of their training, that is, the student would continue to work for the government for a length of time equivalent to the time they received government support. Furthermore, appointment to consultant posts would follow from successful completion of the full programme. Lastly, it was anticipated that the new consultants would have a significant leadership role in the future development of pathology in Zambia. The programme would be deemed successful if at least two to three students graduated each year, were retained within the public sector in Zambia and assisted with the training of future students.

### Recruitment of students

To recruit students, the School of Medicine placed an advert in one of the local newspapers for Zambian doctors interested in pursuing MMed training in pathology at the University Teaching Hospital, Lusaka. The main criteria for acceptability were the possession of a basic medical degree at the University of Zambia (or equivalent), registration with the Health Professionals Council of Zambia, and completion of at least the intern year and one year as a senior house officer, or equivalent, in country of previous practice. Applicants who satisfied these criteria were interviewed by the course tutors to assess and confirm commitment before selection.

### Programme

The programme, exams and assessments were conducted under the School of Medicine, University of Zambia, regulations for postgraduate degrees. The students had to complete five courses over the four-year programme (Figure 1). Progress from one course to the next was dependent on passing the end-of-course exam or assessment. The University of Zambia requires external examiners for end-of-year exams in year 1 and for the final exams in year 4; Professor Dhiren Govender from the University of Cape Town, South Africa, and Professor Martin Hales from the University of Witwatersrand, Johannesburg, South Africa, filled this role 2011–2013 and 2014–2016.

Students had a weekly programme of tutorials alongside practical sessions and group teaching, the latter taking place around the microscope in the pathology department in the University Teaching Hospital, Lusaka. The great majority of the teaching was done by local pathologists, but some sub-speciality teaching was provided by teachers visiting from the United Kingdom for approximately two weeks (e.g. liver, gastroenterological and urological disease and forensic pathology). These teachers were all volunteers, recruited on the basis of being long-standing consultants with experience of teaching trainee United Kingdom staff. It was hoped to involve trainee staff from the United Kingdom, but this proved not possible (see section ‘Lesson learned’). Sessions on general skills such as leadership and management, ethics and professionalism (as provided for all the university’s MMed students) were interspersed throughout the programme. A review of the curriculum and programme was undertaken in 2015, which confirmed that the programme was successful and that only minor changes to timing were needed.

### External clinical placements

Clinical placements outside Zambia in the third year of tuition were an integral part of the programme. They provided trainees with the opportunity to experience techniques and specialisms with limited availability in Zambia – liver, renal, neuro-muscular and bone and joint biopsies and excisions, specialist cytology and autopsy cases and specialist techniques like immune histochemistry and molecular biology. In 2015 and 2016, the clinical placements took place in the Toronto General Hospital, Toronto, Canada, under Professor Runjan Chetty, and to the Aga Khan University Hospital in Nairobi, Kenya, under Dr Shahin Sayed and Dr Zahir Moloo.

Through these placements, the trainees acquired new clinical and service development skills. The experience enhanced not only their clinical practice, but also their personal development, as they were exposed to new ways of working in different environments. In addition, the clinical placements encouraged regional collaboration through the exchange of ideas, of lessons learned and through exchange and sharing of resources. They also gave trainees opportunities to attend international conferences while abroad.

### Costs

We do not have access to the costs incurred by the Zambian government, University of Zambia and the University Teaching Hospital, Lusaka, but the costs covered by the THET grant from 2009 until the end of 2015 are shown in Table 1.

**TABLE 1:** Tropical Health Education Trust costs for development of MMed programme in Zambia, 2010–2016.

Cost category	Cost	Description
Short-term and mid-term trip	£25 447.00	Short-term and long-term volunteer trips
Pathology laboratory overhead budget	£83 747.40	Share of office rent, staff salaries, etc.
Hilditch equipment	£3198.72	Microscopes bought at auction for pathology laboratory, University Teaching Hospital, Lusaka
Wirsam equipment	£14 227.00	Multi-head microscope
Pathology student – Clinical placements	£72 731.00	Clinical placements in Nairobi and Toronto up to December 2015
Curriculum and programme review	£23 367.50	Curriculum review, mileage and expenses, and invigilation trips
**Total cost**	**£221 718.62**	**-**

### Training outcomes

After a further one-year preparation period, the MMed in pathology was launched in 2011 with four recruits. The first final exam was held in October 2015 and three out of the four candidates passed. In October 2016, another one of two candidates passed. Thus, in the first two cycles of the programme, the number of local pathologists in Zambia had increased from 4 to 8. All these graduates are currently (2018) working within the University Teaching Hospital, Lusaka. Two have joined the Medical School as lecturers and one is working under the Ministry of Home Affairs as a forensic pathologist. He is currently training in Toronto, Canada, on a year’s forensic pathology fellowship. The fourth graduate has remained in the employ of the Ministry of Health. Currently (2018) there are two students in year 4, five in year 3, one in year 2, and two in year 1 (Zambia graduates about 100 medical students per year).

## Lessons learned

### Challenges

As one would expect in setting up a new postgraduate programme anywhere, there were a number of challenges and many lessons were learned. However, there were a small number of key challenges (Box 1). One of the most significant, somewhat unexpected and difficult to address, was the differing perspectives of the Ministry of Health and of the Ministry of Education. This became especially apparent on issues of funding where trying to identify whether funding for any particular need arose from a service demand or an educational one. Assigning an activity to one or the other was often impossible. Indeed, the apprenticeship nature of pathology (and most medical) education, where a student is taught while performing routine service, means that trying to separate the costs of the two activities is, by its very nature, impossible. This meant that some activities were on the boundaries of the Ministry of Education and the Ministry of Health, with neither willing to fund the activity. This ministerial split responsibility occasionally also contributed to difficulties in identifying an appropriate decision-maker. On these occasions, decisions on key issues were passed back and forth between ministries and sometimes never completely resolved. This split was also replicated at hospital and university level and even within the pathology department.

BOX 1 Main challenges in developing a MMed programme in Zambia, 2010–2016.

- Differing perspectives of the Ministry of Health and the Ministry of Education
- Local decision-making process sometimes obscure
- Funding for space, books (including journals, ebooks, ejournals), equipment (e.g. microscopes)
- United Kingdom contributions: problems with leave of absence, recruitment of United Kingdom trainees
- Visa and registration requirements in countries hosting trainees on external placements

Given the constraints on funding, resources were inevitably another challenge. The biggest constraint was provision of space for the students for private study. Funding for significant new building was primarily the responsibility of the university and the Zambian government and became a prime example of the divergence of opinion between the respective ministries. Even modest refurbishment became difficult as the student numbers increased with each year’s recruits. Obtaining books, journal subscriptions and microscopes, although requiring considerable effort, was less difficult due to the generosity of charities and well-established international organisations dedicated to providing educational support to LMICs. Funding the visits of the external visitors was also largely not an issue as the THET grant covered these costs. Funding the clinical placements was initially well covered by the THET grant, but as numbers increased this became more difficult. In addition, inevitably there were costs associated with the placement that had not been anticipated. For example, in the original budget, we did not allow for fees for the host departments of the clinical placements, necessitating some last-minute budgetary reorganisation (deferred spending and cuts). Similarly accommodation for the trainees abroad proved more expensive than anticipated.

The overall spending on the THET grant over the six years was £221 718.62. Accordingly, the cost of each graduate in the first five years of the programme was £55 429.00 ($71 464.00 at current rates). The two biggest costs were the clinical placements and the overheads for the administration of the programme. Most of the rest of the costs were travel and the curriculum review undertaken in 2015. Given that these components had substantial overseas involvement, this is perhaps not surprising. In the future, with much less overseas involvement, the costs per graduate will diminish substantially. Similarly, recruitment of more students in each year will reduce the cost per student.

An unexpected issue was the difficulties of United Kingdom staff obtaining time off work to visit and teach. Employers proved very reluctant to view such activity as good for their institution, despite the argument that the returning staff would likely be refreshed with broader horizons. Most visitors had to use holiday entitlement, which inevitably reduced the flexibility and numbers of possible contributors. Similarly, it proved unexpectedly impossible to recruit trainees from the United Kingdom. Originally, we had intended to recruit United Kingdom pathology trainees towards the end of their five-year training programme. It was hoped that, having been through a similar experience recently, they might have a greater insight into the challenges faced by the new Zambian trainees. In addition, like their senior colleagues, it was hoped that the experience would broaden their horizons and benefit their later careers. The recruitment difficulty appeared to be related to constraints within the postgraduate training programmes that had been recently introduced in the United Kingdom where time out from the highly structured programme requires official permission, including formal approval that the external environment is appropriate for training. While this is not unreasonable, it introduced a layer of complexity and bureaucracy which may have deterred some potential participants. Another factor may have been the fact that not all the time spent in Lusaka would have been recognised as equivalent to training time in the United Kingdom, resulting in some extension in the duration of trainee’s overall training. Perhaps not unexpectedly, dealing with the bureaucracy of obtaining visas and registration with the local medical board for the Zambia students while on their clinical placements took more time than anticipated.

### Recommendations

Firstly, to overcome the inevitable problems of starting a new training programme, it is crucial to have both broad ownership and commitment (Box 2). This must be a programme owned and desired by the local staff, university and the national government. The latter two organisations are crucial as resolving issues in a national programme cannot be dealt with by an external or overseas organisation. It is also necessary to have a clear leader and champion who can deal with the issues as they arise. This may seem obvious, but it bears emphasis as implementation of the programme would simply have been impossible otherwise.

BOX 2 Programme development recommendations.

- Broad, local ownership and commitment with local champion, leader or driver is crucial
- Adequate flexible funding necessary
- Effective external driver or leader important
- Regular (if infrequent) face-to-face meetings necessary
- Effective, regular, reliable communication vital
- Effective local administration important
- Collating information on possible resources would be useful
- Long-term planning (> 10 years) is vital
- Exit strategy crucial

Secondly, adequate funding is obviously crucial, but it also needs to be flexible and relatively easily increased – if only modestly. Starting a new programme means that underestimates in budgeting will probably be made, either due to unfamiliarity with costs or because unexpected needs arise – for example, the need to find fees for the clinical placements. Budgetary flexibility is needed to address these unexpected costs. Knowledge of the numerous international charities and organisations that provide free or low-cost access to educational materials is invaluable. A collated list of such organisations would be ideal.

Thirdly, in a hybrid programme with both local and external participants, the local leadership needs to be matched with an external leader and champion. The challenges of distance and resource constraints mean there needs to be a clear external individual who is willing and able to take on the challenges. This person needs to be a pathologist. For example, dealing with the problems of identifying external visitors would have been almost impossible via a committee or a non-pathologist. Similarly, it is difficult to imagine identifying and getting the collaboration of external departments and examiners as a non-pathologist. Additionally, it is necessary to have effective external administration. Dealing with several funding bodies, ensuring compliance with appropriate financial and other regulations, identifying external teachers and organising their participation over several years cannot be done without effective administration.

Fourthly, in setting up any new programme, there are always problems and regular (e.g. weekly) effective communication between participants is vital. This is particularly true in a hybrid programme such as the Zambian one. When the programme was initiated, even email was somewhat intermittent, proving frustrating. This has improved greatly in recent years with better telecommunication methods such as Skype. It is also necessary to have regular, if infrequent, face-to-face meetings. These are not so much to solve problems (although annual review of progress and appropriate modification to the programme is best done face-to-face) but to provide opportunities for participants to develop relationships and build trust.

Lastly, it is crucial to recognise that initiating such a hybrid programme is not a short-term commitment. To allow the programme time enough to stabilise requires at least 5 years and perhaps up to 10 years. This is particularly important to ensuring appropriate continuity of funding and external support. Conversely, it also means ensuring that there is a jointly agreed exit strategy for the external support to allow the local participants sufficient time to prepare for independent ownership.

### Discussion

Of the various issues discussed above, two stand out.

The first is that the single most difficult and intractable issue was getting the Ministry of Health and the Ministry of Education to agree on which would fund activities and infrastructure that had both educational and service aspects. Such a split between university and hospital perspectives is not unique to Zambia. It is found in most countries where medical education and service delivery are the responsibility of different authorities and is similarly contentious. However, where resources are severely constrained, the tensions are magnified.

Given this, what are possible solutions? Once a programme is running, there is no obvious solution to this problem other than repeated discussions with the key individuals – this is where strong local leadership is vital, as identifying such individuals needs local insight. In retrospect, a discussion of the issue during the preliminary stages of setting up the programme, resulting in an agreed allocation of responsibilities, would have been the most appropriate solution. For instance, agreeing during the planning phase to create a joint fund which could have then been used by the programme organisers to fund such combined activities and infrastructure would have saved considerable time, effort and angst. Whatever the approach, the key is to have the issue clearly identified and addressed early in the planning phase.

The second issue is that in many low-resource regions, it is very difficult, if not impossible, for any one country to set up and sustain a fully comprehensive training programme. For example, in any one country, there may just not be the necessary number of trainers or the required case mix. The creation of the Zambia MMed in pathology would have been impossible without sharing resources between countries, in this case, between Zambia, the United Kingdom, Canada, Kenya and South Africa. However, the use of trainers from the United Kingdom and of placements in Canada markedly increased the costs of the programme.

This is where a regional body, such as a college like COPECSA, could have a key role – especially where such institutions are seen as dedicated to standards and independent of any particular country or government policy. As a transnational organisation, COPECSA could have identified, for example, sites with specialist staff and equipment necessary for specific investigations. It could then have facilitated the short-term transfer of trainees who need training in such topics to this site – this would be analogous to, but much cheaper than, the external clinical placements to Toronto of the Zambia programme. Similarly, having a COPECSA regional core curriculum would have been of major benefit in the creation of the Zambia MMed, saving time and effort. Lastly a regional body such as COPECSA could also have reduced the burden of designing and undertaking examinations by recruiting regional examiners and sharing assessment procedures. A general benefit of such activity would be to ensure high and equivalent standards across the region, perhaps leading in due course to trans-regional recognition of qualifications.

### Conclusion

The creation of a postgraduate training programme in pathology in countries with limited local resources has many challenges but taking a hybrid approach involving the sharing of resources with other countries is a pragmatic and feasible solution. It is likely that such hybrid programmes are more achievable and sustainable if the countries involved are part of a regional grouping and are facilitated by an independent body such as COPECSA.

## References

[1] Wilson ML, Fleming KA, Kuti MA, et al Access to pathology and laboratory medicine services: A crucial gap. Lancet. 2018;391(10133):1927–1938. https://doi.org/S01406736(18)304586 29550029 10.1016/S0140-6736(18)30458-6

[2] Sayed S, Cherniak W, Tan SY, et al Improving pathology and laboratory medicine in low income and middle income countries: Roadmap to solutions. Lancet. 2018;391(10133):1939–1952. https://doi.org/S01406736(18)304598 29550027 10.1016/S0140-6736(18)30459-8

[3] Republic of Zambia National Health Strategic Plan 2006–2010 [homepage on the Internet]. Ministry of Health. [cited 2019 Oct 26]. Available from: http://www.zukhwa.ed.ac.uk/sites/default/files/alth_Strategic_Plan_Revised_July_20__2006_1_.pdf

[4] Busisiwe CM, Mwesigwa S, Mboowa G, et al The collaborative African genomics network training program: A trainee perspective on training the next generation of African scientists. Genet Med. 2017;19:826–833. 10.1038/gim.2016.177 28383545 PMC5509501

[5] Zulfu AA, Abass SK, Awadalla H, et al Assessment of pathology trainees’ satisfaction: results of a survey from Sudan. J Public Health Emerg 2018;2:29–35 10.21037/jphe.2018.10.01

[6] Nelson AM, Hale M, Diomande MIJ-M, et al Training the next generation of African pathologists. Clin Lab Med. 2018;38:37–51. 10.1016/j.cll.2017.10.004 29412884

[7] Sayed S, Mutasa R, Kaaya E, et al Establishing COPECSA – The Regional ECSA College of Pathology. Afr J Lab Med. 2020;9(2):a979 10.4102/ajlm.v9i2.979 PMC7276350 32537427

